# Aurophilic Molecules on Surfaces. Part I. (NapNC)AuCl on Au(110)

**DOI:** 10.1021/acsomega.3c02473

**Published:** 2023-08-08

**Authors:** Michael Györök, Thorsten Wagner, Petra Gründlinger, Uwe Monkowius, Peter Zeppenfeld

**Affiliations:** † Institute of Experimental Physics, Surface Science Division, Johannes Kepler University, Altenberger Straße 69, 4040 Linz, Austria; ‡ School of Education, Johannes Kepler University, Chemistry, Altenberger Straße 69, 4040 Linz, Austria

## Abstract

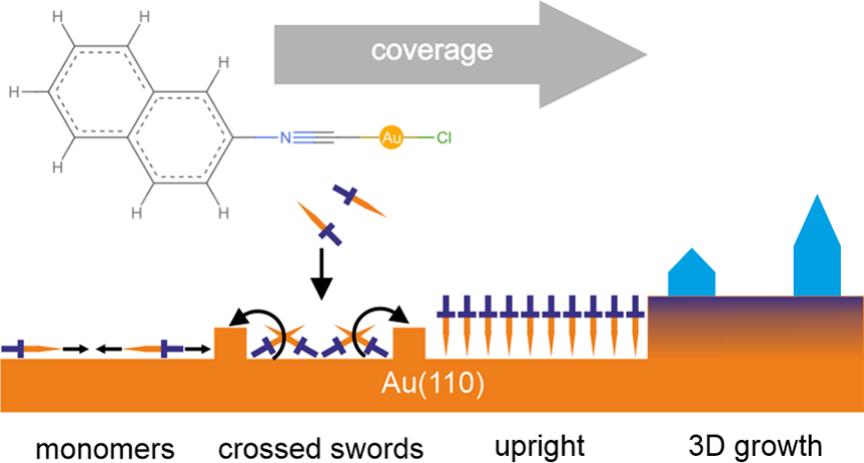

Aurophilicity is a well-known phenomenon in structural gold chemistry and is found in many crystals of Au(I) complexes. However, these attractive dispersion forces between and within complexes containing Au(I) moieties have not been well studied in ultrathin films. In this paper, we elucidate the interaction of chlorido(2-naphthyl isonitrile)gold(I) on and with Au(110) surfaces. Already during physical vapor deposition, the condensation of ultrathin films is monitored by photoelectron emission microscopy (PEEM) and by incremental and spectrally resolved changes in the optical reflectance (DDRS). Additional structural data obtained by STM and LEED reveal that the “crossed swords” packing motif known from the bulk is also present in thin films. The molecular arrangement changes several times during thin-film deposition.

## 1 Introduction

The engineering of highly organized systems fabricated from molecular building blocks opens up new perspectives for the control of matter and applications in nanodevices. In this context, self-assembly of organic molecules on surfaces is an important concept, which allows studying the interaction of molecules with their environment from a more fundamental point of view. In addition to the interaction between the adsorbates and substrate, the intermolecular interaction is also of great importance. By tuning the latter, it is possible to force the molecules to form certain patterns—e.g. one-dimensional stacks or two-dimensional networks.^1^ The interaction between the molecules can be localized on only a part of the molecule but does not need to be directional. Therefore, the self-assembled structures are most commonly based on van der Waals interactions (to the substrate) and H-bonds or, in general, noncovalent bonds (between the molecules). In two consecutive articles, we want to show a new path: the formation of molecular patterns driven by aurophilic interaction.

In 1988, Schmidbaur and co-workers introduced the term “aurophilicity” or “aurophilic attraction” for attractive interactions between Au(I) atoms in its complexes.^2−5^ The oxidation of gold atoms yields the closed-shell configuration [Xe][4f^14^][5d^10^]. At first glance, one might expect a mutual repulsion of the gold atoms due to their positive ion charge. However, after the appearance of many structural data of Au(I) complexes in the solid state, it became clear that Au(I) atoms attract each other if the steric situation (i.e., small ligands) allows for an effectual approach of the gold atoms. Due to a flat energy profile, the range of distances considered as aurophilic attraction is relatively broad. Therefore, as a rule of thumb, Au–Au distances below 0.35 nm can be assumed to be aurophilic bonding (see refs (6−8) and references therein), although this value is above twice the van der Waals radius, i.e., 2*r*_vdW_ = 0.332 nm.^9^ Because of the closed-shell configuration, these attractive interactions cannot be based on covalent bonds but rather on particularly strong dispersion interactions. This is also confirmed by thorough quantum mechanical investigations. These unusually strong dispersion interactions are a result of the strong relativistic effects exerted by the gold atom. Thus, there is an abundance of examples of Au(I) compounds, whose arrangement in the solid state appears to be governed by aurophilicity.^7,10,11^ Furthermore, it has been found that these attractive interactions are not limited to Au(I), but can also be found for other ions with closed-shell configuration, which is why they are commonly referred to as “closed-shell interactions”.^12−14^ Because relativistic effects are most pronounced in gold, it also exhibits the strongest attractive forces, reaching values up to those of hydrogen bonds. Metallophilic bonding of other closed-shell ions is much weaker.

**Figure 1:**
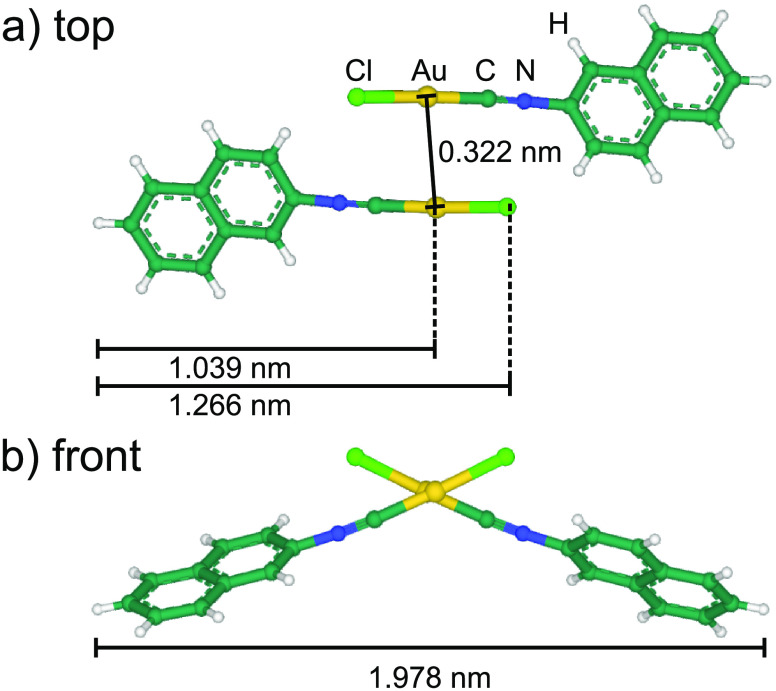
(a) Top view and (b) front view of a (NapNC)AuCl dimer aligned as “crossed swords”. The position of the atoms were taken from ref (20). For visualization, Mol* was used.^21^

So far, only a few studies have been carried out on the aggregation of gold complexes on surfaces.^15^ This is particularly surprising, since Au(I) complexes are used as precursors for vapor deposition methods.^16−19^ In the first part of this study, we report on the growth of chlorido(2-naphthyl isonitrile)gold(I) (Figure 1) on an anisotropic Au(110) surface. In part 2, the experiments on an isotropic Au(111) surface will be presented. However, due to the electronic ground state of the pure gold surfaces, no aurophilic interaction between the molecules and the substrate is to be expected. Note: although the IUPAC classification suggests [AuCl(NapNC)] as an abbreviation, we will use (NapNC)AuCl hereafter to be consistent with previous publications.^15,20^

The crystal structure of the molecule is known from a single-crystal X-ray structure analysis.^20^ This study also shows that the molecules preferentially form a dimeric structure that can be described as “crossed swords” (see Figure 1). There are 8 molecules in the unit cell of the studied crystals. The dimers change their orientation from one to the next in two dimensions. The shortest distance between the gold atoms is 0.3224 nm, which is less than twice the van der Waals radius of gold (*2r*_vdW_ = 0.332 nm).^9^ From this, the presence of significant aurophilic interactions in the solid state structure can be assumed.

## 2 Experimental Details

The gold complex (NapNC)AuCl was synthesized according to ref (20). All experiments were performed in an ultrahigh-vacuum system with a base pressure in the range of about 5 × 10^–10^ mbar. Besides a Focus PEEM with integrated sample stage and imaging energy filter (retarding field), the vacuum system houses an Omicron VT-AFM and an Omicron Specta-LEED. For the excitation of the photoelectrons, a Xe lamp (PEEM movies and differential reflectance spectroscopy during deposition) or a He I lamp (Focus HIS13 during PEEM spectroscopy) were used.

Prior to the deposition experiments, the Au(110) single crystal was cleaned by repeated cycles of Ar ion sputtering (Ar^+^ energy 900 eV, current density *j* ≈ 2.5 μA cm^–2^, angle of incidence ≈45°) for 30 min and subsequent annealing to 800 K at a rate of 1 K s^–1^.

**Figure 2:**
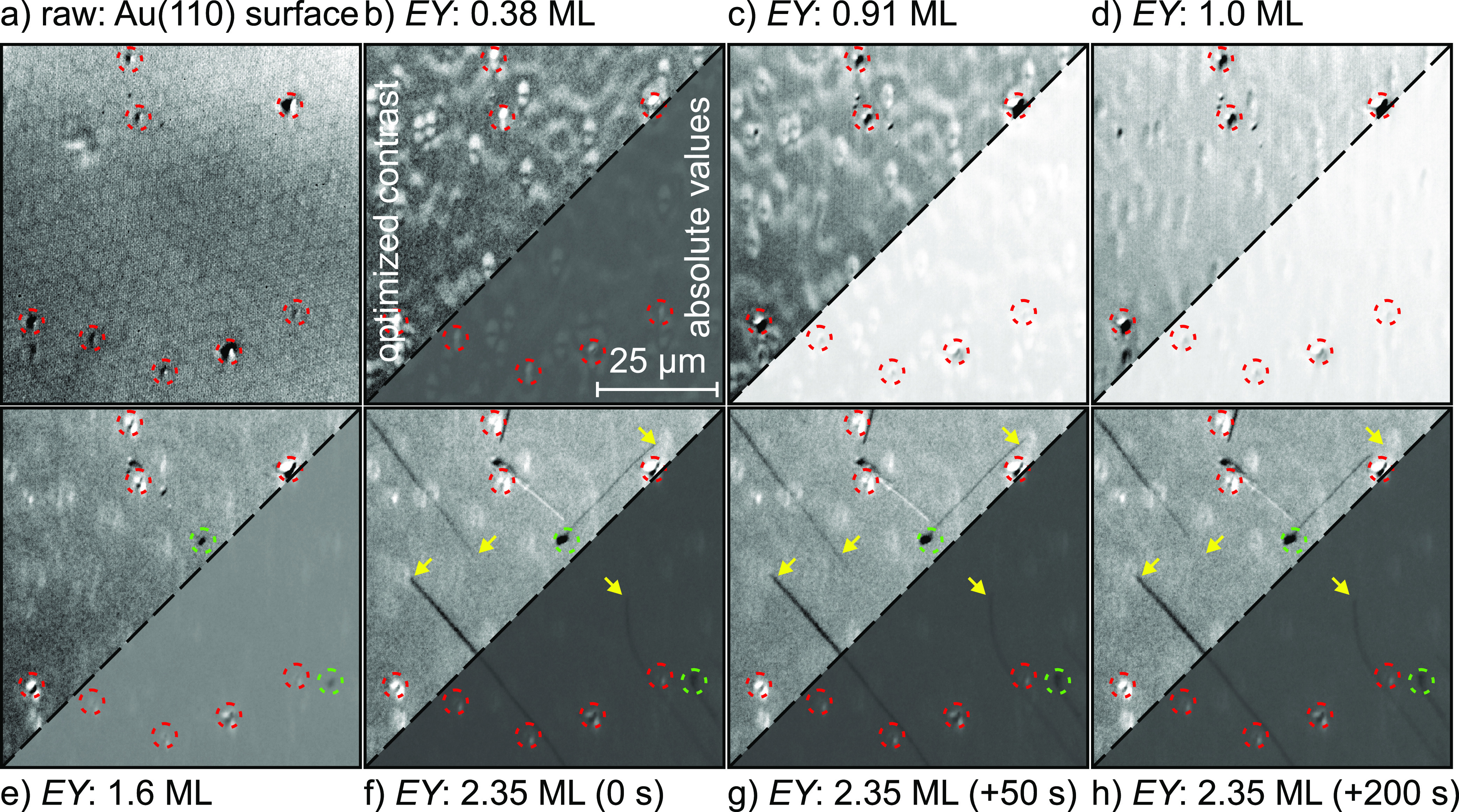
PEEM images with a size of 75 μm × 75 μm: (a) Average of 40 images before opening the shutter of the evaporator; (b–h) images normalized according to eq 1 representing the local variation of the electron yield at the indicated coverages. The lower right corner of the images uses a common gray scale allowing a comparison of the changes in local electron yield. The upper left corner shows the region of interest with an individually optimized contrast to emphasize the variations within each image. The red dashed circles mark point defects already being present on the bare surface. The green dashed circles highlight (2D or flat 3D) islands of (NapNC)AuCl on the surface. In the later stage of growth, longer needle-like crystallites form. The yellow arrows mark their tips upon closing the shutter in image (f). Images (g) and (h) thus show the surface 50 and 200 s after stopping the deposition, respectively.

During physical vapor deposition of the (NapNC)AuCl molecules from a quartz crucible held at a constant temperature of 403.15 K via a PID controller (ventiotec OVD3), PEEM images were taken every 5 s. Figure 2a shows a PEEM image of the surface before the deposition. Although the pixel noise (due to the Poisson statistics) is greatly reduced by averaging 40 images, this “raw” image shows several artifacts related to the detection system: (i) individual channels of the MCP as well as pixels of the Andor Neo camera have slightly different amplification/conversion factors (hot or dead pixels), (ii) a background structure (honeycomb pattern) due to the arrangement of the MCP channels, (iii) inhomogeneous illumination with the Xe lamp, vignetting due to apertures and lenses (in the electron and photon optics), and structures burnt into the screen/MCP, and (iv) an offset of the camera (dark counts). The dark counts can be easily corrected by subtracting a “dark” image (*D*) recorded without any illumination. The contributions i–iii can be described as a variation of the gain/conversion factor across the image (background *B*). Therefore, we divided all images *I* by the image shown in Figure 2a. As a result, the bare surface is given by a structureless image with intensity values of around 1. Any changes to this value are mainly proportional to the variation of the local electron yield (EY) as a function of time *t* and coordinates *x*, *y*.
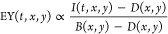
1

Such normalization of the images bears the risk that lateral variations within the image like point defects, step bunches, and so on are not taken properly into account. Therefore, we have marked the most visible defects in the series of images shown in Figure 2.

**Figure 3:**
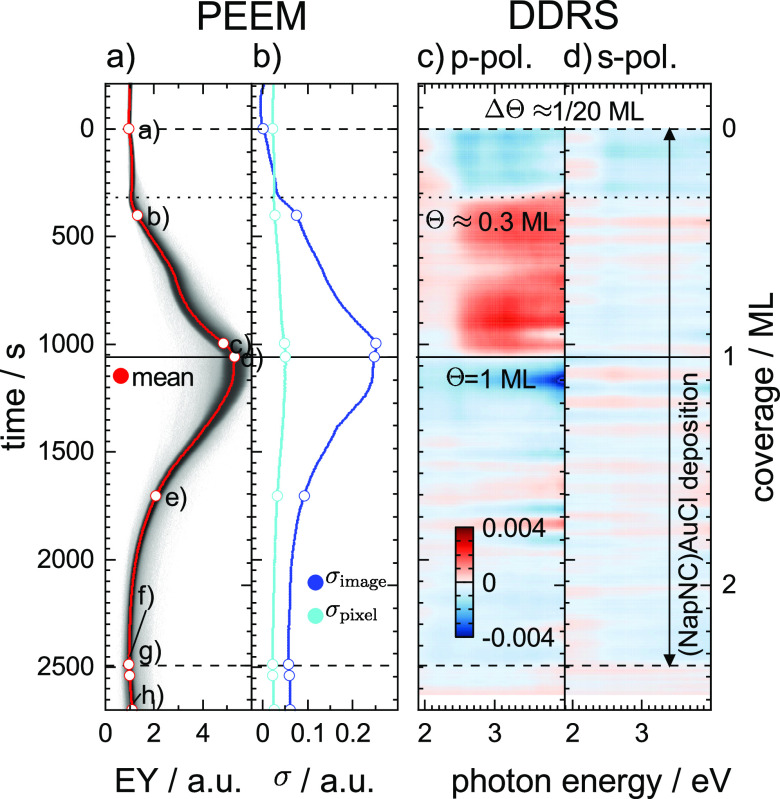
(a) Histograms of the electron yield (EY) of the individual PEEM images shown as rows in a nonlinear gray scale representation. The mean electron yield (MEY) is plotted as red dots. (b) Standard deviation related to the pixel noise (σ_pixel_) and the inhomogeneity of the images due to the morphology (σ_image_) calculated from eq 4. The open circles in (a) and (b) mark the data points related to the images shown in Figure 2. DDRS calculated according to eq 5 for (c) p- and (d) s-polarized light. In all graphs, the left axis represents the time since the shutter. The right axes represent the corresponding coverage, assuming a constant deposition rate.

Besides the distribution of the electron yield (shown as false color encoded background in Figure 3a) we also extracted the mean electron yield MEY
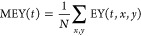
2and the estimate of the variance
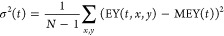
3

Here *N* denotes the number of pixels within the respective region of interest.

In contrast to ref (22), we follow here a different approach to separate the contribution to the variance originating from the pixel noise (Poisson distribution) and the image inhomogeneity. As stated already above, the pixel noise is reduced by averaging over *n* consecutive images (with just incremental changes in the morphology). Assuming a Poisson distribution for the pixel noise, it follows that
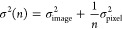
4which allows separating the two contributions by averaging images. Details about the method will be published elsewhere.

As described in ref (23), simultaneously and synchronized to the acquisition of PEEM images the changes in optical reflectance can also be measured for s- and p-polarized light (pol-DRS). For PEEM imaging and differential reflectance spectroscopy, the same Xe lamp was used with the light beam directed at an angle of 65° with respect to the surface normal. During the evaporation process, the spectral intensity *S*(*hν*,*t*) measured after reflection at the sample surface increases or decreases, depending on the optical properties of the evaporated molecules and the film thickness. Assuming a constant intensity of the incoming light, these signals can be related to changes in reflectance, i.e., *R*(*hν*,*t*) ∝ *S*(*hν*,*t*). After the reflection of the light at the sample surface, the beam is split into a p-polarized and an s-polarized part by a Glan–Thompson prism and is focused by a lens into two separate spectrometers (STS-UV from Ocean Optics). The spectrometers span a spectral range from 190 to 650 nm (corresponding to photon energies between 6.52 and 1.91 eV), but due to the transmission of the viewports and other optical components, there is almost no detectable intensity above *hν* = 4 eV. The spectral resolution is 1.5 nm. In Figure 3c,d, we show only the incremental changes in the optical data following the definition of the so-called DDRS signal:^23^
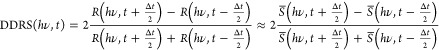
5

Here, Δ*t* is the time interval needed for the deposition of approximately 1/20 monolayer. The overline above the spectral intensity *S* indicates an averaging of all spectra in the intervals (*t* – Δ*t*/2; *t*[ and ]*t*; *t* + Δ*t*/2), respectively, to improve the signal-to-noise ratio.

## 3 Results and Discussion

Figure 2 shows selected PEEM images acquired during the deposition of (NapNC)AuCl on a Au(110) surface. From the same data set, also the distribution of the electron yield (EY) and its mean value (MEY) have been extracted as well as the standard deviation related to the pixel noise (σ_pixel_) and the inhomogeneity of the images due to the morphology (σ_image_) (see Figure 3). Besides some point defects, the bare surface is structureless (see the raw image in Figure 2a). During the initial deposition, no structure is visible on the normalized images (representing the changes in EY). However, point defects are decorated. Their contribution has almost no effect on the MEY value, and only σ_image_ increases slightly. There is a steep increase of σ_image_ at about Θ = 0.3 ML, which coincides with the appearance of a lateral inhomogeneity in the PEEM images shown in Figure 2b. Simultaneously, the mean electron yield increases steeply up to 5 times the value of the clean surface.

We associate the maximum of the MEY after a deposition time of 1060 s with completion of the first monolayer: usually, the first molecular layer has the largest effect on the changes in work function due to the direct contact between the adsorbate and the substrate. For illumination with a Xe lamp, the photoelectrons mostly originate from the substrate. Since the excited photoelectrons have to pass through the organic layer before leaving the sample surface, they are efficiently scattered after the completion of the first monolayer. Consequently, the electron yield monotonically decreases with increasing film thickness.^24^ Initially, dark spots (2D islands or platelet-like crystallites) and later elongated dark needles appear in the PEEM images indicating a Stranski–Krastanow growth.^25^ 2D island formation and 3D crystallite growth result in the expected attenuation of the photoelectrons by the organic material.^24^ Taking into account both effects, i.e., the changes in work function and the photoelectron attenuation, it is reasonable to assume that the maximum of the electron yield marks the completion of the monolayer. As discussed later, the maximum of the electron yield coincides also with an abrupt change of the pol-DDRS signal shown in Figure 3c,d.

The recovery of image homogeneity at this point is a final argument: the standard deviation related to the image homogeneity reaches a maximum at about Θ = 0.91 ML, i.e., 0.09 ML before the maximum of the MEY. From Figure 2c,d the variation of the photoelectron yield across the field of view is reduced. The pattern now seen in the normalized images will not disappear upon further deposition. Therefore, MEY exhibits a short plateau starting at θ = 1 ML.

Upon further deposition of (NapNC)AuCl, the mean electron yield MEY and the standard deviation σ_image_ both decrease continuously. At a nominal coverage of about Θ = 1.04 ML dark islands appear in the PEEM images (highlighted with green circles in Figure 2e). Their positions are not correlated with the visible defects on the surface, and their number and size increase slowly until about Θ = 1.6 ML. In fact, we find just two such islands in the 75 μm × 75 μm large field of view shown in Figure 2. The area with dark spots is definitely smaller than one would expect from the nominal coverage, indicating either (i) a higher packing density in 2D islands of the second layer or (ii) 3D growth. In both cases, the condensed structure is in equilibrium with a 2D molecular gas phase (or structures smaller than the resolution limit of the PEEM, which is about 150 nm).^22^ The increasing density of this gas phase is therefore the main reason why the MEY decreases continuously.

The dark islands and the previously mentioned defects on the surface can act as nucleation centers for needle-like structures, which appear above a surface coverage of Θ = 1.6 ML. Again, only a negligible part of the surface is covered by these structures, so we assume that the needles are taller 3D crystallites. These needles have preferred orientations with respect to the substrate, indicating a defined epitaxial relation between the wetting layer and the 3D crystallites on top.

The total amount of deposited (NapNC)AuCl on the Au(110) surface in Figures 2 and 3 corresponds to Θ ≈ 2.35 ML. When the shutter of the evaporator is closed, the morphology still undergoes significant changes: during the first 10 s the needles extend in length and the image intensity of the wetting layer increases slightly (see MEY in Figure 3a). This indicates the presence of a 2D gas phase in the second layer. As the molecules are incorporated into the needles, the density of the gas phase decreases so that the photoelectrons originating from the substrate are less scattered.

Within the next 150 s, we observe that most needles shrink again; see the yellow arrows in Figure 2 marking the tips of the needles at the moment when the shutter was closed. Some of them become even shorter than they had been when the deposition was stopped. This behavior could be due to Ostwald ripening.^26^ We cannot confirm with our data that larger needles become longer so that it is most likely that the dissolved material extends the needles in either their width or height.

Figure 3 also shows the changes in optical reflectance, which were acquired simultaneously with the PEEM images. Due to the polarizing beam splitter in the path of the reflected light, we can separate the s- from the p-polarized reflectance. Whereas the s-polarized light is just sensitive to a transition dipole moment parallel to the surface, the p-polarized light also contains information about the out-of-plane component.

We first discuss the reflected light with p-polarization. Upon opening the shutter, there is a decrease of the optical reflectance for *hν* ≥ 2.5 eV. This is where two optical transitions (one at *hν* = 2.5 eV and one at *hν* = 3.5 eV) were observed for the bare Au(110)(1 × 2) surface by reflectance difference spectroscopy (RDS).^27^ Therefore, we may argue that the electronic structure of the (1 × 2) reconstructed surface changes due to the interaction with the adsorbed molecules. According to ref (20), absorption features for (NapNC)AuCl are expected only above *hν* = 4 eV, which is just outside the spectral range that can be reliably detected with our setup.

Exactly at that time, when the MEY starts to increase steeply (Θ ≈ 0.3 ML), the sign of the DDRS spectrum changes (for *hν* ≥ 2.5 eV). Now, the incremental change of the reflectance is positive until the monolayer coverage is reached. At this point, the incremental change stops abruptly until a negative change occurs at the moment when the dark islands appear in the PEEM images (Θ ≈ 1.04 ML). Based on the optical absorption data for (NapNC)AuCl published in ref (20), we assume that the maximum of the optical feature lies above 4 eV (and is therefore not visible in Figures 3c,d).

In principle, the 3D structures (islands and needles) should have a negligible contribution to the DDRS signal since the surface area covered by these structures is quite small. However, the sudden condensation of the islands could considerably reduce the density of the 2D molecular gas phase and, thus, create a significant change in the DDRS signal.

The s-polarized light shows a tiny signal only in the coverage range between 0 ML and about 0.3 ML, which is already a factor of 4 smaller than the p-polarized signal but has similar spectral characteristics. This can be interpreted in such a way that in this coverage range the relevant transition dipole moment is mainly oriented in the surface plane, while for coverages between 0.3 and 1 ML the effects are mostly associated with an out-of-plane component.

### 3.1 Submonolayer Structure

**Figure 4:**
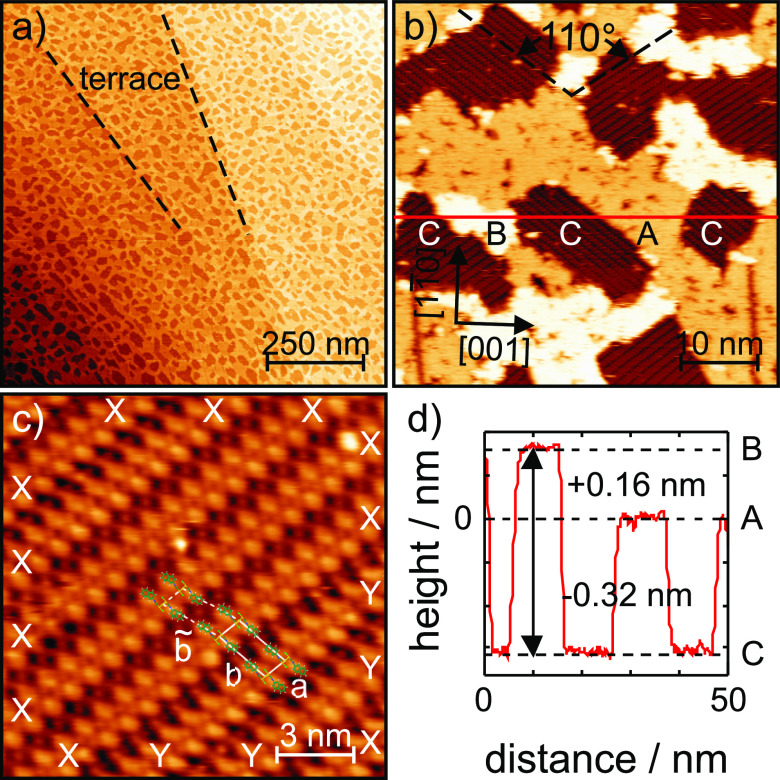
STM data of a Au(110) surface covered with about 0.3 ML of (NapNC)AuCl: (a) survey scan showing that step edges of the Au terraces are overgrown by molecular islands (1 μm × 1 μm, *I*_*T*_ = 10 pA, *U*_sample_ = 0.8 V); (b) detail scan and d) corresponding cross-section (red line) revealing three distinct apparent heights marked with A–C (50 nm × 50 nm, *I*_*T*_ = 10 pA, *U*_sample_ = 0.8 V); (c) STM image with molecular resolution. Dimer rows marked with X and Y exhibit two different shifts relative to each other (15 nm × 15 nm, *I*_*T*_ = 1 nA, *U*_sample_ = −0.8 V).

The STM image in Figure 4a shows a 1 μm × 1 μm large region of the Au(110) surface. The deposition of 0.3 ML (NapNC)AuCl results in an irregular pattern of darker areas surrounded by brighter ones. The areas appearing darker in the STM are about 10 nm in diameter. Several terraces of the substrate (with a width of about 200 nm) are visible in the image. The step edges between the terraces seem to be overgrown by molecules.

Figure 4b represents a close-up of such structures. The red line marks the position of the height profile shown in Figure 4d. Three height levels (A–C) can be distinguished. The differences between these levels are determined to be Δ*Z*_AB_ = +0.159(15) nm, Δ*Z*_BC_ = −0.470(26) nm, and Δ*Z*_AC_ = −0.315(14) nm. We want to emphasize that different images were used for the evaluation, and mean values and their standard deviation are given here, so that the sum of Δ*Z*_AB_ and Δ*Z*_AC_ agrees with Δ*Z*_BC_ only within the statistical uncertainty.

For the Au(110) surface, the theoretical layer spacing is 0.144 nm. Taking into account the statistical spread of the data as well as the uncertainty of the STM calibration (estimated to be about 5%), we can assume that areas B are exactly one gold monolayer higher than areas A. We were unable to resolve any atomic (or molecular) structure within regions A and B. In general, they appear somewhat fuzzy with some darker, fringed defects. This appearance is typical of only weakly bound adsorbates in a dilute phase (2D molecular gas). The regions C correspond to surface areas covered with a condensed structure of (NapNC)AuCl as shown in Figure 4c.

We used the flooding function of the software WSxM^28^ to mask the different regions A–C and to determine the relative coverage of the three. Table 1 summarizes the results for the image shown in Figure 4b.

**Table 1:** Coverage Θ of the Three Structures A–C Identified in Figure 4b^a^

coverage	expected	measured
Θ_A_	0.475	0.46(3)
Θ_B_	0.175	0.19(5)
Θ_C_	0.350	0.35(4)

a The flooding function of WSxM was used to determine the structures.^28^

From the series of PEEM images, we deduced a (NapNC)AuCl coverage equivalent to 0.3 ML. This is in very good agreement with Θ_C_ obtained from the STM image. Areas A correspond to the original reconstructed Au(110)-(1 × 2) surface (“missing row”). If molecules condense on the Au surface, its reconstruction is lifted and the excess gold atom forms an additional layer (B) on top of A. These areas B are also (1 × 2) reconstructed but on average one gold layer higher than A. Due to expulsion of the Au atoms upon condensation, areas B and C should exhibit a ratio of 1:2. In fact, the measured areas are in very good agreement with this model (see Table 1). The gold regions (A and B), however, cannot be resolved atomically but appear fuzzy due to the presence of a dilute 2D molecular gas phase on top of the still reconstructed Au areas, so we can suspect that not all molecules are visible.

It can be seen from Figure 4b that the condensed molecules in the areas C arrange into rows with orientations ±55° with respect to the  direction of the Au(110) substrate. This points to the fact that there exists two mirror domains, in which the molecules stack along one of the two diagonals of the unit cell of the bare, unreconstructed surface, i.e., along the  directions.

Figure 4c shows some more details. The molecular rows are formed by stacks of (NapNC)AuCl dimers. Such dimers are visible as two protrusions in the STM image, which are probably associated with the naphthyl groups. Along each row, the dimers are periodically spaced at .

One can distinguish two relative arrangements of dimers across adjacent rows. (i) The dimers can be “in-phase” aligned, corresponding to the −XX– and −YY– configurations in Figure 4c. (ii) If the rows are “out-of-phase”, there is a lateral offset by half a unit cell, i.e., a⃗/2. In Figure 4c, this corresponds to the −XY– or −YX– configurations.

Taking into account the findings by STM and by LEED (shown in the Supporting Information), the superstructure for 0.3 ML (NapNC)AuCl on Au(110) in the −XY– configuration can be described by a commensurate structure with an epitaxy matrix
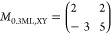
6

Based on the data for the bulk crystal of (NapNC)AuCl published in ref (20), such an arrangement in alternating dimer rows is closer to the projected bulk structure (bulk_III_—see Table 2) than the arrangement corresponding to an “in-phase” sequence, namely −XX– or −YY– given by
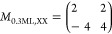
7

**Table 2:**
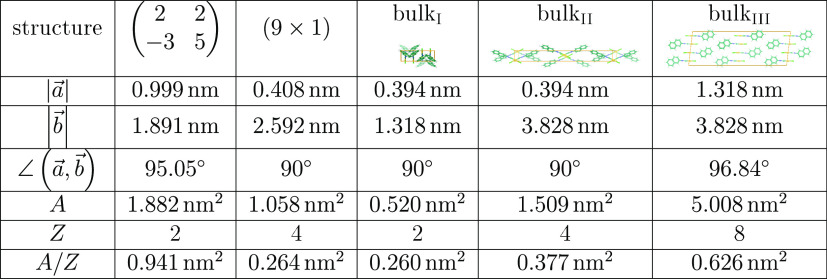
(NapNC)AuCl Structure on Au(110) and 2D Projects of the Bulk Structre^a^

a The 2D projections of the bulk structure are based on the data published in Ref (20). a⃗ and b⃗ denote the unit cell vectors. *A* is the area of the unit cell, and *Z* is the number of molecules within the contact plane.

The model assumes a dimeric structure of (NapNC)AuCl on the surface, in which the naphthyl groups lie nearly flat on the surface. In addition, we assume a fixed orientation of the “crossed swords” formed by the two NCAuCl groups of a dimer with respect to the substrate.^15,20^ In Figure 4c, we tentatively chose an orientation of the dimer within the unit cell on the surface, which corresponds to that in the 3D bulk structure.

(NapNC)AuCl itself is achiral but becomes chiral upon adsorption on the surface.^29,30^ In particular, the “crossed swords” dimers are chiral if they lie flat on the surface. In principle, a left- and right-handed arrangement in the dimer is possible, but the asymmetric shape of the unit cell just allows one enantiomer within the respective unit cell due to steric repulsion. Only in the mirror domains—see Figure 4b—will the dimers have the opposite handedness.

### 3.2 Monolayer Structure

**Figure 5:**
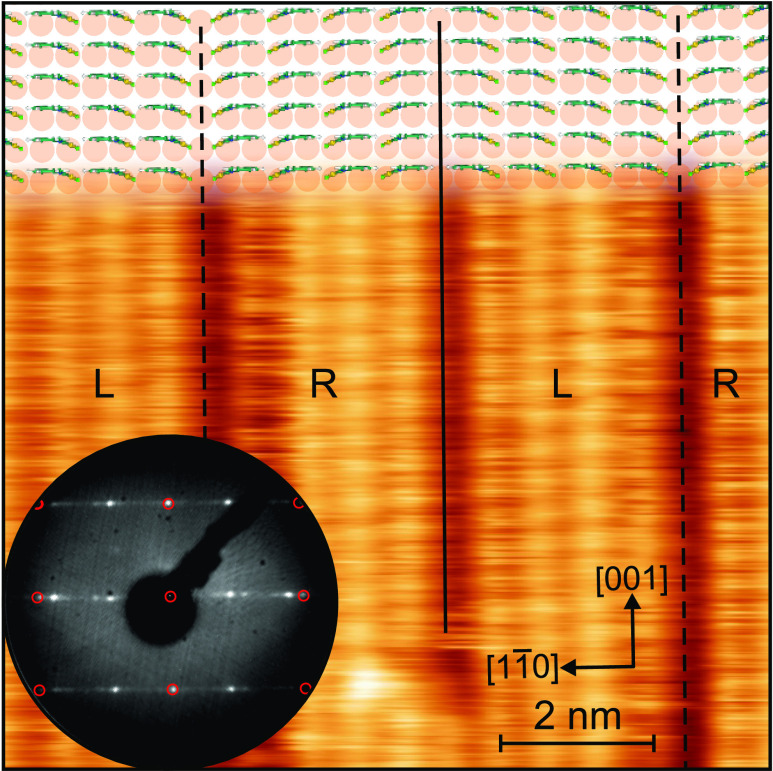
STM image showing an area of 10 nm × 10 nm on the Au(110) surface covered with about 1 ML (NapNC)AuCl. After deposition, the sample was annealed at 353 K for 5 min. Imaging parameters: *U*_sample_ = 0.8 V and *I*_*T*_ = 5 pA. The fast scan direction was from left to right. A preliminary structural model of four molecular rows spanning nine substrate unit cells is shown in the upper part of the image. Due to the alternating handedness (tentatively labeled with L and R), the molecules form domains with different boundaries (L–R and R–L) indicated by the dashed and solid black lines, respectively. The inset at the bottom shows the LEED pattern of the same sample acquired with an electron energy of *E*_kin_ = 48 eV. The expected positions of the diffraction spots originating from the (unreconstructed) Au(110) substrate are marked with red circles.

In another experiment, we stopped the deposition slightly after reaching the maximum of the PEEM transient. In this case, the (NapNC)AuCl coverage should be just a little bit higher than that of a full monolayer (1 ML). Only upon annealing the film to 353 K have we been able to acquire molecularly resolved STM images such as the one shown in Figure 5. The annealing temperature was chosen far below the one that we used for the crucible during physical vapor deposition, so that we can exclude any sizable desorption of molecules upon annealing.

The diffraction pattern shown in the inset of Figure 5 clearly indicates a high degree of order on the surface. The periodicity along the [001] direction is identical to that of the bare, unreconstructed Au(110) substrate; i.e., the (1 × 2) missing-row reconstruction has totally disappeared. The STM image shown in the same figure reveals rows of protrusions with an identical contrast along the [001] direction. Therefore, we can safely assume that the molecular structure is commensurate with this direction.

Along the orthogonal  direction a periodic arrangement is found in the STM: two, three, or even four rows of molecules form domains, which are interrupted by more fuzzy and wider domain walls (see vertical lines in Figure 5). It seems that the tip of the STM can pull molecules out of these domain walls since the fuzziness is influenced by the scan direction. Some of the domain boundaries like the one marked with a solid line in Figure 5 appear less fuzzy.

The LEED pattern shows spots along the  direction at the positions expected for the bare substrate and additional spots with a spacing of 1/9 of this distance. This is consistent with the STM image in Figure 5, where an average of four molecular rows form a domain spanning 9 substrate spacings along the  direction. However, some LEED spots are brighter than others due to a nontrivial structure factor of the unit cell as well as the form factor of the individual molecules. More details of this discussion can be found in the Supporting Information. For the following discussion, we assume the structural model shown at the top of Figure 5: a (9 × 1) commensurate unit cell containing four molecules.

It may be surprising that the rows do not densely cover the surface, but between them (more or less), periodically arranged wider troughs can be found. There are two fundamental scenarios. (i) The actual coverage is less than a close-packed monolayer. Simple but unlikely reasons are that not enough molecules were deposited initially or part of them was desorbed during the annealing. A reorientation of the molecules due to a phase transition into a denser phase could also cause free areas on the surface. (ii) The molecules cannot pack more densely due to repulsive interactions. A reason could be again that the molecules are chiral on the surface. This causes steric repulsion between the two possible enantiomers.

A reorientation of the molecules upon monolayer completion can be inferred from the molecular densities in the low versus high coverage phase: In contrast to the *M*_0.3ML,XX_ superstructure with almost flat-lying (NapNC)AuCl molecules (see Table 2), the density of protrusions in the *M*_1.0ML_ structure is more than 3 times higher. The high density (or small size) of the protrusions is no longer in agreement with (almost) flat-lying molecules. Therefore, we conclude that each protrusion represents a single, almost upright-standing (NapNC)AuCl molecule as shown at the top of Figure 5. It is not clear whether the Cl or the naphthyl group faces downward to the surface. The structures seen in the STM images might also be consistent with upright-standing dimers in which the naphthyl group would be imaged by the STM.

The reason we cannot explicitly exclude dimers becomes obvious from Table 2 is that the surface unit cell of the *M*_1.0ML_ structure corresponds nicely to about half of the projected bulk unit cell (bulk_I_). The bulk unit cell consists of an alternating sequence of “crossed swords” dimers facing in opposite directions. The STM data suggest that there is just one orientation of the (NapNC)AuCl molecules within each domain. There is no indication that adjacent rows exhibit alternating orientations of the molecules or dimers. Just the existence of the domain walls and the fuzzy behavior of some molecules at these boundaries may point to the fact that molecules with different handedness might be present on the surface. If the (NapNC)AuCl molecules are anchored by the AuCl moiety to a Au atom of the substrate, a certain degree of freedom for the molecules may result: an individual molecule may rotate and tilt around this anchor. Within rows of such standing molecules, at least the rotation could be prohibited. Therefore, we assume that only molecules/dimers with the same orientation condense in a domain formed by the well resolved (unfuzzy) rows in Figure 5.

Because of steric repulsion, rows with opposite orientations of the molecules cannot come as close as rows, in which the molecules have the same orientation. This allows the STM tip to pull out individual molecules as it scans across them. This leads to fuzzy boundaries between the ordered domains with alternating orientation of the molecules (L vs R). In addition, our results show that the domain boundaries L–R and R–L have a slightly different contrast (see solid and dashed lines in Figure 5). Due to the asymmetry of the molecules, different parts of the molecules face each other at these domain boundaries.

We emphasize that the drawings of the molecules in Figure 5 do not represent the outcome of a geometry optimization of a single molecule on the surface. The chosen arrangement resembles half of a dimer as published in ref (20): (NapNC)AuCl is slightly bent out of the plane given by the naphthyl group. We anticipate perfectly planar molecules if they do not form “crossed swords” dimers.

## 4 Comparison with the Bulk Structure

Table 2 summarizes our experimental results: For low coverages (Θ = 0.3–0.6 ML), we were able to identify by STM and LEED rows of dimers on the surface, which form a  superstructure. This corresponds well to a 2D projection of the bulk unit cell published in ref (20), which we refer to in Table 2 as bulk_III_. The bulk unit cell with a total of 8 molecules belongs to the *C*2/*c* space group. This reduces the *C*1 point symmetry on the surface: there is only one energetically preferred orientation of the dimers on the surface. In addition, the dimers in the bulk crystal exhibit different “heights”. On the surface, the coordinate normal to the surface is the same for all dimers; see STM images shown in Figure 4. As a result, the length  reduces roughly to half of this value, namely a spacing between dimer rows of 1.891 nm. Neglecting the 3D orientation of the molecules, the second axis would reduce to . In fact, the distance measured in Figure 4 is  and thus is larger by a factor of 1.5. This can be explained by the fact that identical lattice sites are energetically preferred: if the dimers would stack as in the bulk crystal, the structure would no longer be simple commensurate but rather include dimers at alternating, nonequivalent adsorption sites.

An even better agreement is found for the (9 × 1) superstructure, which dominates close to the completion of the monolayer, and the projected bulk_I_ phase. This arrangement implies a reorientation of the molecules: instead of lying almost flat on the surface, the molecules here stand upright. It is not surprising that this phase does not appear for low coverages (Θ < 0.6 ML), since this rearrangement removes the overlap of the molecular π electron system with the surface. This is expected to be energetically costly and has to be compensated for by the newly formed π–π interaction between the molecules. However, the reorientation leads to a higher density of molecules on the surface, i.e., a smaller footprint of each molecule (see *A*/*Z* in Table 2).

The reorientation of the molecules can also be monitored by their optical signature. Only the out-of-plane component of the optical transition dipole moment contributes to the signal measured with p-polarized light; an in-plane optical transition dipole moment would contribute in both polarizations to the measured DDRS signal. Actually, Figure 3 shows a characteristic change at Θ ≈ 0.3 ML. We interpret this abrupt change with the formation of “crossed swords” dimers on the surface. At low coverage, the molecules adsorb as monomers. They lie mainly flat on the surface due to the van der Waals interactions of the naphthyl group with the surface. We expect for the measured spectral range just an almost constant in-plane contribution of the monomers to the dielectric function, because we are far below the optical transitions characteristic for the molecules.^20^ In addition, the optical transitions of the molecules are quenched due to the interaction with the electronic system of the substrate. Such flat-lying monomers are expected to induce only a small change of the work function consistent with the PEEM transient (Figure 3a).

At about 0.3 ML the density of molecules on the surface reaches a critical point: the condensation into solid structures formed by “crossed swords” dimers starts. These dimers certainly have an out-of-plane component: (i) the “crossed swords” configuration itself implies a tilting of the molecules and (ii) within the dimers, the molecules are no longer flat but slightly bent. Consequently, the p-polarized component of the reflected light is most affected, which now leads to a positive increment as seen in Figure 3c. The reorientation of the molecules due to dimer formation also implies a major redistribution of charges at the solid–vacuum interface. Consequently, the electron yield measured with PEEM increases steeply once dimer formation starts.

## 5 Summary and Conclusions

During physical vapor deposition of ultrathin (NapNC)AuCl films on Au(110) surfaces, several stages can be distinguished in the experiments performed with PEEM, DDRS, STM and LEED: (i) initially, adsorption of the molecules as monomers on the surface forming a 2D molecular gas phase, (ii) formation of dimers with a “crossed swords” configuration driven by the aurophilic interaction, (iii) (simultaneous/consecutive) condensation into 2D islands accompanied by the lifting of the (1 × 2) Au reconstruction via expulsion of Au atom, (iv) a reorientation transition upon further deposition of nearly flat-lying molecules, forming “crossed swords”, to (almost) upright standing molecules, similar to the packing in the bulk crystal but possibly more triggered by a π–π interaction of the naphthyl groups and a bonding of the AuCl moiety to the substrate than by “pure” aurophilic attraction, and (v) Stranski–Kastranow growth mode for thicker films followed by some relaxation of the film after stopping the deposition. The data suggest that not only are single molecules deposited by physical vapor deposition but also the “crossed swords” dimers, indicative of aurophilic attraction, are subsequently formed on the surface and that this interaction is not quenched by the interaction with the surface.

## Supporting Information Available

The following data are available free of charge. The Supporting Information is available free of charge at https://pubs.acs.org/doi/10.1021/acsomega.3c02473.

Work function changes upon deposition and additional structural information including STM and LEED data as well as detailed structure models (PDF)
