# Supplementary material for: Aurophilic Molecules on Surfaces. Part I. (NapNC)AuCl on Au(110)
Source: ACS Omega. 2023 Aug 8;8(33):30109–17. doi: 10.1021/acsomega.3c02473 (PMC10448646; doi:10.1021/acsomega.3c02473)
Supplement: Supplementary file 1 — ao3c02473_si_001.pdf [file ao3c02473_si_001.pdf]

Aurophilic Molecules on Surfaces - Part I: (NapNC)AuCl on Au(110) –supporting information–

Michael Györök,† Thorsten Wagner,*,† Petra Gründlinger,† Uwe Monkowius,‡ and Peter Zeppenfeld†

†*Johannes Kepler University, Institute of Experimental Physics, Surface Science Division, Altenberger Straße 69, 4040 Linz, Austria*

‡*Johannes Kepler University, School of Education, Chemistry, Altenberger Straße 69, 4040 Linz, Austria*

E-mail: thorsten.wagner@jku.at

1 Work Function Changes

To measure the work function changes upon deposition of (NapNC)AuCl on Au(110), the excitation source of the PEEM was replaced with a He(I) gas discharge lamp, which supplies photons with energy $h\nu = 21.2\,\mathrm{eV}$. The kinetic energy of the photoelectrons emitted from the surface was varied by means of a retarding potential, such that only the photoelectrons above a variable threshold energy are detected in the PEEM (energy filter). In this way, we have recorded photoelectron emission spectra (Figure S1a) for selected coverages of (NapNC)AuCl. The work function W of the system can then be calculated via

$$W = h\nu - (E_{sco} - E_F) \tag{S1}$$

Here E_{sco} denotes the secondary cut-off and E_F the Fermi edge in the spectra. We assume that E_F does not change upon deposition of (NapNC)AuCl. Therefore, the work function change ΔW can be solely determined by the shift ΔE_{sco} of the secondary cut-off.

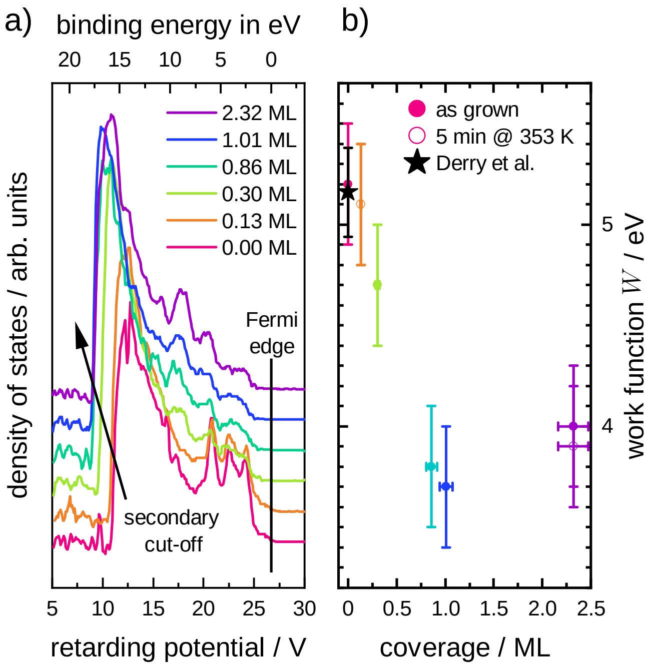

Figure S1: (a) Photoelectron spectra for different coverages obtained from the retarding potential dependence of the mean image intensity. (b) Evolution of the work function W as a function of the coverage Θ. For reference also the value reported by Derry at al. (see ref 1) for Au(110) is included. The solid symbols represent the ultrathin films as grown. The open symbols are used for samples, which were annealed at 353 K for 5 min. The color code is the same in both graphs.

The value listed by Derry et al.[1] for the work function of Au(110) is 5.12(22) eV. The evaluation of our data yields 5.2(3) eV for the work function of the bare Au(110) surface. This confirms that our data reproduce even the absolute value and not just the relative changes in work function within the experimental uncertainties. In our case, the latter are estimated to 0.3 eV due to the uncertainty in determining the Fermi edge and the secondary cut-off and the discrete steps of 0.1 eV between the data points while ramping the retarding potential.

The experiments show a significant decrease of the work function with increasing coverage to a minimum at around 1 ML (see Figure S1b). Towards higher coverages (2.32 ML in

Figure S1b), the work function increases again, but slightly.

2 (NapNC)AuCl Structure

The structural characterization of the samples with STM (see Figures 4 and 5 of the main paper) were supplemented by LEED studies. A compilation of the various LEED patterns observed at different coverages of (NapNC)AuCl is shown in Figure S2. In particular for a coverage of 0.6 ML several well-defined LEED patterns were found. Besides the (9×1) domain wall phase (see discussion below) and the $\left(\begin{smallmatrix} 2 & 2 \\ -3 & 5 \end{smallmatrix}\right)$ superstructure, we could identify a third structure, which was not confirmed by STM. In general, the samples exhibit strong local variation of the LEED pattern if moving them in front of the LEED.

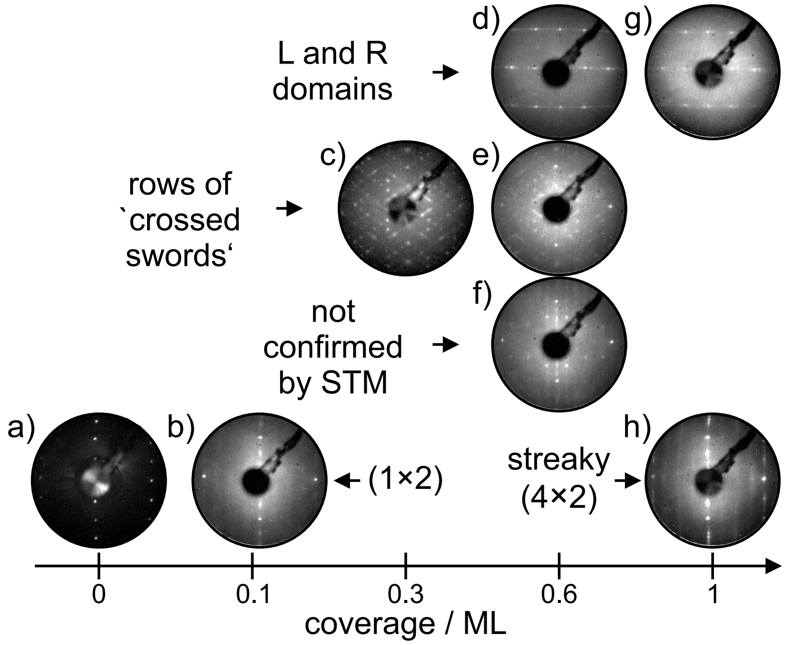

Figure S2: Survey of LEED images before and after deposition of (NapNC)AuCl onto a Au(110)(1×2) surface. The energy of the primary beam was $E = 48\,\text{eV}$ for all images.

To get more insight into the structural details, we have simulated the LEED patterns for various arrangements of molecules according to the kinematic theory.[2] To this end, we

describe the incoming electrons of energy E by a plane wave

$$A_i = A_0 \cdot e^{i\vec{k}_0 \cdot \vec{r}} \tag{S2}$$

with amplitude A_0 and wave vector

$$k_0 = \frac{\sqrt{2\,(E - V_0)\,m_e}}{\hbar^2}. \tag{S3}$$

Here, $\hbar$ denotes the (reduced) Planck constant, m_e the mass of the electron, and V_0 the so-called 'inner potential'. Although the (complex valued) inner potential is important to predict the correct energy dependence of the intensity of the LEED spots (IV-LEED, see refs 3 and 4), we can neglect it in the framework of the kinematic theory and the fact that our simulations deal with purely 2D structures, only.

Assuming n scatterers at positions $\vec{r}_n$, the superposition of the elastically scattered waves in direction $\vec{k}_f$ yields the diffracted intensity

$$I\left(\Delta\vec{k}\right) \propto \left|\sum_{n=1}^{N} f_n \cdot e^{i\Delta\vec{k}\vec{r}_n}\right|^2 \tag{S4}$$

$\Delta\vec{k} = \vec{k}_f - \vec{k}_i$ is the momentum transfer between the incoming ($\vec{k}_i$) and scattered wave ($\vec{k}_f$) and f_n is the so-called atom form factor of the n-th scatterer. For purely elastic scattering, $k_i = k_f = k_0$ holds. The sum in eq S4 has to be taken over all scatterers within the so-called transfer width. Due to the low coherence of the incoming electron beam, we assume a transfer width of 15 nm in our simulations. In the following, we will describe each molecule as a single scatterer and neglect the contribution of the individual atoms in the molecule.

2.1 0.3 ML to 0.6 ML

Figure 4 in the main paper shows a survey of the STM data obtained for a (NapNC)AuCl coverage of $\Theta = 0.3$ ML. A corresponding LEED pattern is shown in Figure S2c. The

experimental findings can be explained by the model shown in Figure S3, which we will discuss in detail in the following.

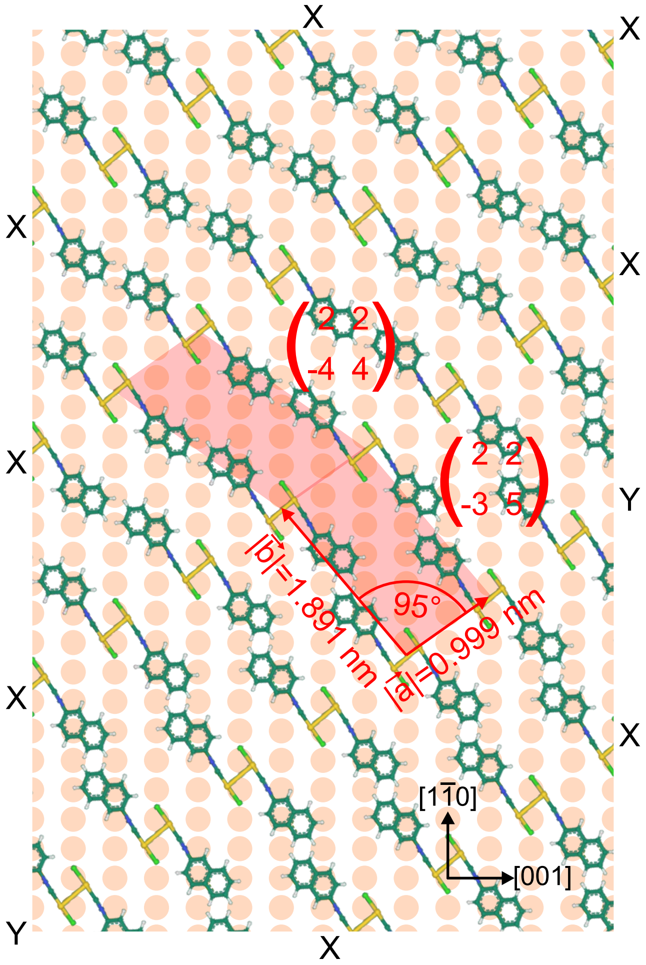

Figure S3: Model of the -XX- stacking ('in phase') and -XY- stacking ('out of phase') for (NapNC)AuCl on Au(110). The two arrangements differ by the relative shift between adjacent rows along the [1$\bar{1}$1]-direction.

As discussed in the main paper, the superstructure given by the epitaxy matrix

$$M_{0.3\,\mathrm{ML,XY}} = \begin{pmatrix} 2 & 2 \\ -3 & 5 \end{pmatrix} \qquad \text{(S5)}$$

has the highest similarity to the bulk structure and might therefore be the energetically preferred one.[5] In fact, we use the same arrangement of the molecules in Figure S3 as in the bulk structure but just changed the spacing between the dimers. The dimers connected

by aurophilic bonds are aligned parallel to the $\langle 1\bar{1}1\rangle$ directions and the short axis $\vec{a}$ of the unit cell spans exactly two substrate spacings in this direction. In contrast to the bulk unit cell, the stacking of dimers along this direction is not given by a sequence of up and down facing 'crossed swords'. As a consequence, the spacing along this direction is increased due to the steric repulsion of the 'crossed swords' with the same orientation. In fact, the upward pointing 'crossed swords', should be energetically preferred since in this configuration the molecules can interact via the π-electron systems of the napthyl groups with the substrate surface. The commensurate spacing by $\vec{a} = 2\vec{a}_{\mathrm{Au}} + 2\vec{b}_{\mathrm{Au}}$ between the dimers in the $\langle 1\bar{1}1\rangle$ directions further contributes to the stabilization of this adsorption geometry.

The unit cell for an 'in phase' stacking of neighboring dimers rows (-XX- or -YY-) exhibits the same commensurate packing motif along $\langle 1\bar{1}1\rangle$ direction, but a different 'phase' between dimers in neighboring rows:

$$M_{0.3\,\mathrm{ML,XX}} = M_{0.3\,\mathrm{ML,YY}} = \begin{pmatrix} 2 & 2 \\ -4 & 4 \end{pmatrix} \tag{S6}$$

The existence of different stacking motifs suggests a rather weak interaction between the dimers across neighboring rows.

Figure S4 summarizes our simulations of the LEED patterns shown in Figures S2c,e for coverages between $\Theta = 0.3\,\mathrm{ML}$ and $0.6\,\mathrm{ML}$. The top panel illustrates the positions of the scatterers in real space for a single mirror domain within the transfer width of 15 nm. Applying the appropriate symmetry operations according to the p2mm symmetry of the substrate to the diffraction patterns, the simulated LEED patterns including both mirror domains shown in the lower panel of Figure S4 were obtained.

It is striking that none of the structures given by eqs S5 and S6 nor their incoherent superposition can correctly reproduce the measured LEED patterns (shown in Figures S2c,e). Assuming that all sequences of X and Y rows occur with the same probability, we positioned scatterers in a random sequence of X and Y rows as shown in Figure S4c. Here, the

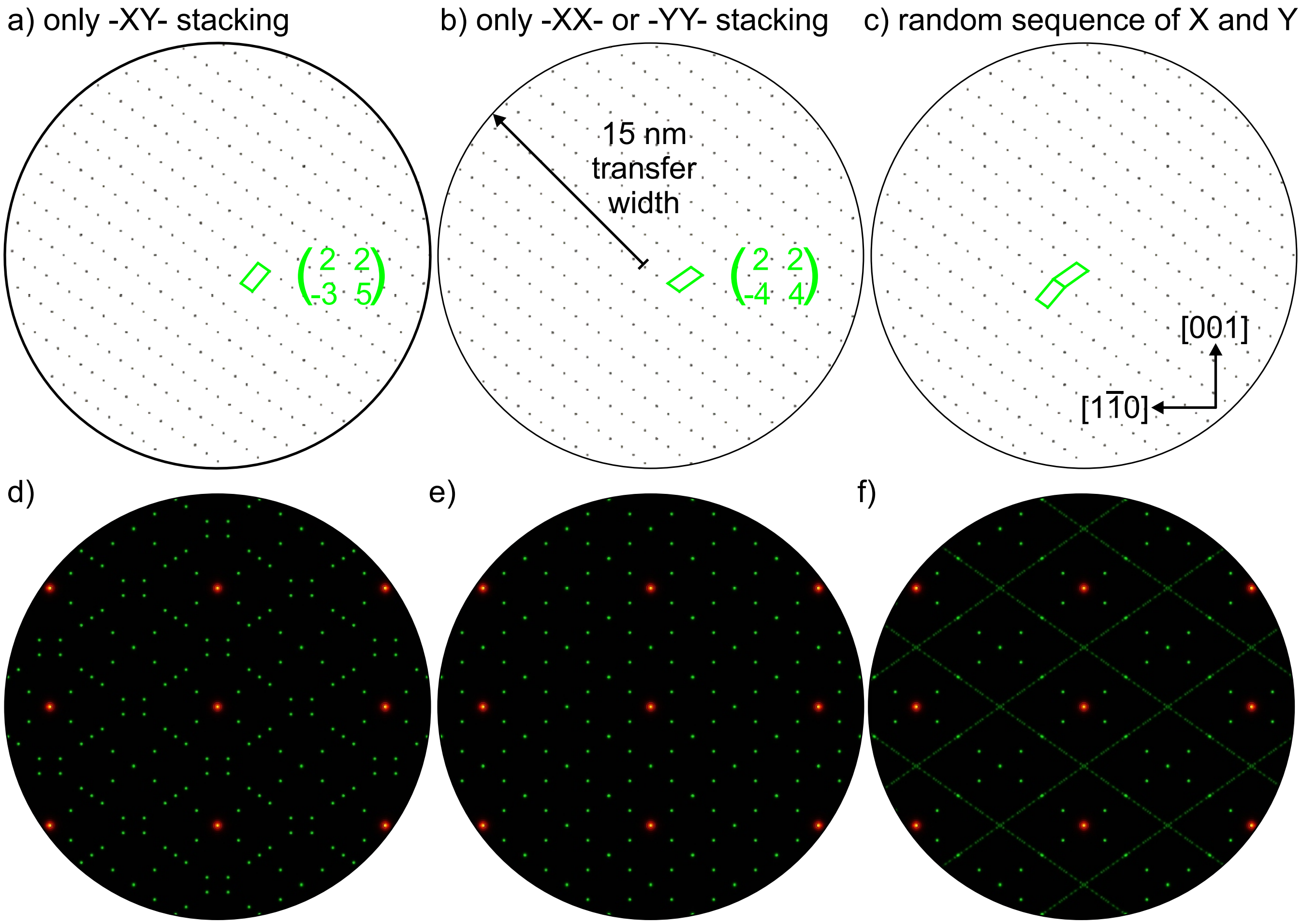

Figure S4: The top panel (a–c) shows the positions of the scatterers in real-space used to simulate the diffraction pattern in the bottom panel (d–f): (a) and (d) periodic arrangement of alternating X and Y rows corresponding to the epitaxy matrix given in eq S5. (b) and (e) periodic arrangement of identical rows X or Y corresponding to the matrix in eq S6. (c) and (f) random sequence of X and Y rows. The kinetic energy of the primary electrons was set to 48 eV. The positions of the LEED spots corresponding to the (unreconstructed) Au(110) substrate are colored in red.

probability of the next row to be of type X or Y was set to 0.5.

As a result, only diffraction spots located on the main 'diagonals' between the diffraction spots of the substrate retain a significant intensity. In fact, only these spots are common in the simulated diffraction pattern of both 'pure' structures shown in Figures S4d,e. All other spots of the 'pure' structures lie on diagonals half way between the main ones, but at different positions. As a result, these diagonals appear as faint lines with a more or less continuous intensity distribution for the random sequence of X and Y rows.

The fact that annealing of the films for 5 min at a temperature of 353 K did not improve significantly the sharpness of the diffraction spots may be taken as an indicator that the interaction between neighboring rows does not greatly affect the adsorption energy. The dominant intermolecular interaction thus occurs along the dimer rows.

2.2 0.6 ML to 1 ML

In the main paper, we discussed the expulsion of atoms upon adsorption of (NapNC)AuCl due to lifting the Au(110)(1×2) reconstruction. In this process, the reconstructed Au islands should be formed on top of the original substrate layer with a total coverage half as large as the surface covered by (NapNC)AuCl. To elucidate this process, we stopped the deposition at $\Theta = 0.6\,\mathrm{ML}$. A corresponding STM image is shown in Figure S5a. As expected, the surface appears rough: although no 'added' islands on the terraces were found, the step edges appear eroded.

The histogram of the STM data displayed in Figure S5b reveals a four level system. The distribution of the heights was fitted successfully with four Gaussian functions, which have all the same width (given by twice the standard deviation). In addition, the four corresponding mean values are almost equally spaced by $\approx 1.1\,\mathrm{nm}$, which is close to the height of a single gold layer.

The survey of the LEED data compiled in Figure S2 also confirms some structural changes: at $\Theta = 0.6\,\mathrm{ML}$, the pattern characteristic for random sequences of X and Y rows

were found, but also the pattern of the later discussed (9 × 1) domains (see below). We even found evidence for a third structure in LEED (see Figure S2f). However, we have not been able to obtain STM images with atomic or molecular resolution in this coverage regime.

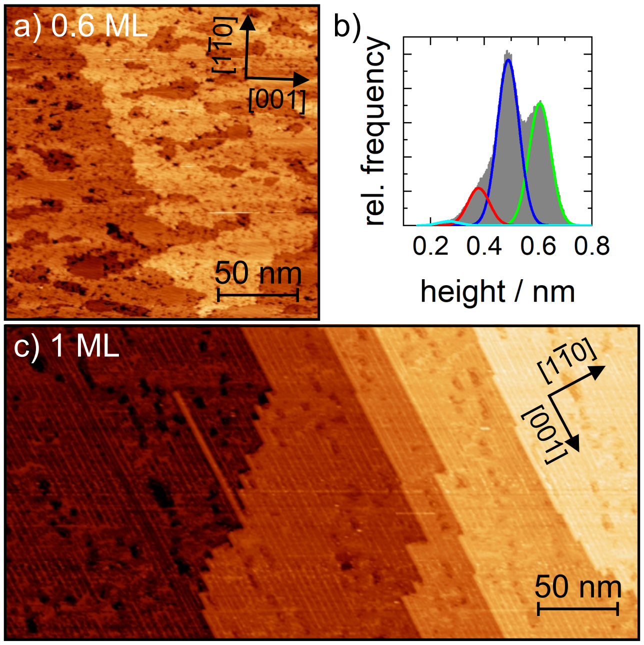

Figure S5: (a) 200 nm × 200 nm large STM scan (I_T = 5 pA, U_{sample} = 0.7 V) of a Au(110) surface after deposition of nominal 0.6 ML of (NapNC)AuCl. (b) Height histogram after careful alignment of the base plane of the STM scan shown in a. The histogram was fitted with four Gaussian peaks. The width of the peaks was constrained to be the same for all. (c) STM image recorded after deposition of nominal 1 ML of (NapNC)AuCl and subsequent annealing for 5 min at a temperature of 353 K. Size: 400 nm × 200 nm, I_T = 10 pA, and U_{sample} = 0.8 V.

Only upon deposition of about 1 ML, such molecular resolution was possible again. Figure S5c shows a larger area and Figure 5 of the main paper some details. Comparing the two STM images in Figure S5 for $\Theta = 0.6$ ML and $\Theta = 1$ ML, it is obvious that the step edges are now straight and follow the direction of the molecular rows. In contrast to the 'crossed swords' phase found at lower coverage with rows along the $\langle 1\bar{1}1 \rangle$ directions, the rows for higher coverages are mainly parallel to the [001] direction of the substrate.

In the following, we want to discuss the LEED pattern, which we identified to be characteristic for the (9 × 1) domains as shown in the STM image of Figure 5 in the main paper. From the STM images, we infer that the most favorable configuration consists of alternating

domains with 4 four molecular rows on average. Indeed, the LEED pattern (see Figure S6d) can be interpreted as a (9×1) superstructure with spots at approximately $n/9$ positions between the substrate spots along the $\left[1\bar{1}0\right]$ direction. Furthermore, the original (1×2) reconstruction of the bare Au(110) surface is lifted by the adsorption of the molecules since the half order spots along the [001] direction are all absent.

Since a domain spans across nine substrate atoms and contains on average 4 rows, the largest distance d between the regular spaced rows is $9/4\, a_{\mathrm{Au}}$. This configuration would result in a high intensity of the $(n \cdot 4/9, m)$ with $n, m \in \mathbb{Z}$ spots along the $\left[1\bar{1}0\right]$ direction. The other spots would have a significant lower intensity.

Depending on the exact adsorption geometry, every value of d between $2a_{\mathrm{Au}}$ and $9/4\, a_{\mathrm{Au}}$ is possible. We even cannot exclude that the distance between the individual rows in a single domain varies within the above range. The fuzziness of the STM images does not allow a more accurate estimate here.

In a next step, we want to include the domain boundaries separating domains containing N rows of the same handedness. In adjacent domains, the molecules have opposite handedness. Keeping the width of a single domain constant at $w = 9\, a_{\mathrm{Au}}$, we have to introduce two more parameters: i.) δ is the shift of the (NapNC)AuCl rows in respect to the Au substrate in $\left[1\bar{1}0\right]$ direction. Here, we allow for $0 \leq \delta \leq a_{\mathrm{Au}}$. In fact, this lateral shift has no impact on the LEED pattern. ii.) For any $d < 9/4\, a_{\mathrm{Au}}$, a non-zero phase shift φ between the rows of adjacent domains results. Having the other parameter fixed, it follows that $\varphi = w - 3nd - \delta$.

The first structure simulated (see Figure S6b) is a regular assembly of four rows in each domain. For the simulation we used $w = 9a_{\mathrm{Au}}$, $d = 17/8\, a_{\mathrm{Au}}$, $\delta = 0$, and $\varphi = 21/8\, a_{\mathrm{Au}}$. Within each domain, the position of the scatterers are given by 0, $17/8\, \vec{a}_{\mathrm{Au}}$, $34/8\, \vec{a}_{\mathrm{Au}}$, and $51/8\, \vec{a}_{\mathrm{Au}}$. The idea behind this is the following: the distance $d = 17/8\, a_{\mathrm{Au}}$ between adjacent rows in a domain is not simple commensurate. In the present case, the fifth row in a domain would be located at $68/8\, \vec{a}_{\mathrm{Au}} = 17/2\, \vec{a}_{\mathrm{Au}}$, i.e., on a local adsorption site shifted by $1/2\, \vec{a}_{\mathrm{Au}}$ with respect to that of the first row. If the first row is adsorbed on a on top site, this would

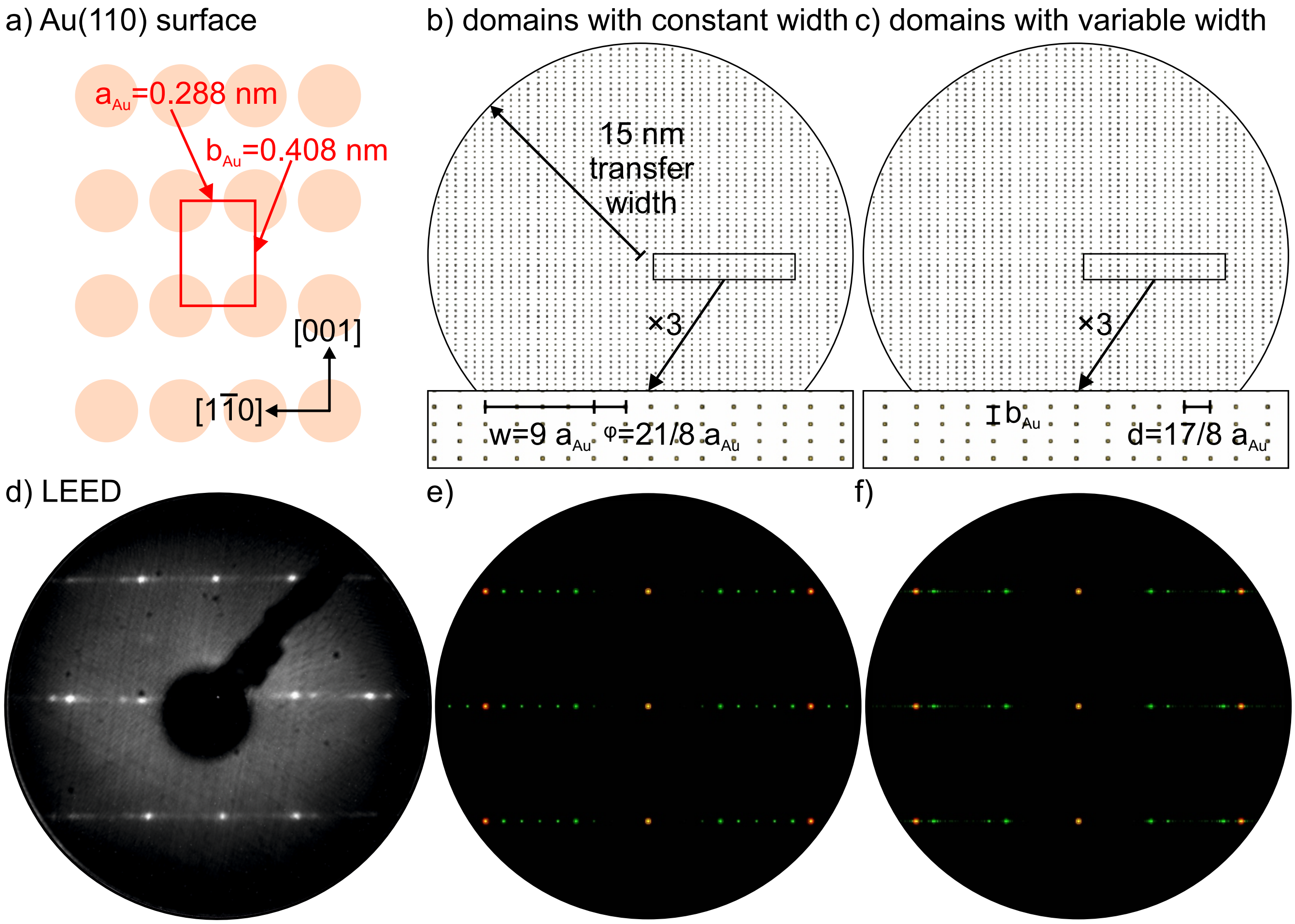

Figure S6: (a) Dimensions of the unit cell of the unreconstructed Au(110) surface used for the simulation of the LEED pattern. (b) regular and (c) random arrangement of rows in real space. (d) The measured LEED pattern was acquired after adsorption of about 1 ML (NapNC)AuCl on Au(110) and subsequent annealing using an electron energy of 48 eV. (e) and (f) show simulated LEED pattern for the arrangements shown in b and c, respectively. The images are a superposition of the diffraction patterns of the unreconstructed Au(110) surface (in red) and the one of the molecular structure (in green). Instead of the complex structure of the molecule only a point scatterer was assumed.

correspond to a bridge-site and vice versa. If the top site is energetically preferred, the bridge site is certainly not. Therefore, the fifth row will avoid it and adsorbs with an additional 'phase shift' of $1/2\,\vec{a}_{\text{Au}}$ starting the next domain.

Figure S6e shows the plot of eq S4 for $k \leq k_{\text{max}} = k_0 \sin 51°$. Here, k_0 is given by eq S3 and the kinetic energy of the electrons used in the LEED experiment, namely $E_{\text{kin}} = 48\,\text{eV}$. The additional trigonometric factor takes into account the opening angle of the LEED screen.

The simulated LEED pattern already reproduces the most prominent features of the measured pattern: the additional spots all fall onto locations corresponding to a (9×1) superstructure, i.e. with a spacing between the superstructure spots in the $\left[1\bar{1}0\right]$ direction, which is a ninth of the one for the unreconstructed Au(110) surface. In addition, the low order spots in the vicinity of the (0,0) spot are almost completely extinct. We want to emphasize that the simulated pattern also exhibits maxima at the integer position of the bare, unreconstructed surface (marked in red). If the spacing between the rows would be equidistant, i.e. $9/4\,a_{\text{Au}}$, these spots would be completely extinct.

The feature that cannot be reproduced by this regular arrangement of domains of equal width is the smeared out intensity along the $\left[1\bar{1}0\right]$ direction. This is an indication of an irregular arrangement of the domain size distribution. Therefore, we tried to account for this in the simulation by modeling the width of the domains using the so-called Γ-distribution:

$$P_{\text{cont.}}\left(X\right) = \frac{X^{\alpha-1} e^{-X/\beta}}{\Gamma\left(\alpha\right)\beta^{\alpha}} \tag{S7}$$

with shape parameter $\alpha > 0$, scale parameter $\beta > 0$, mean value $\mu = \alpha/\beta$, and variance $\sigma^2 = \alpha/\beta^2$. This distribution function was successfully applied to model the diffraction pattern of stepped surfaces.[6] In our context, 'steps' are replaced by 'domain walls'. As the number N of rows in a single domain is not a continuous distribution, we discretize the

probability density according to ref 7.

$$P_{\text{discr.}}\left(N \in \mathbb{N}\right) = \int_{N-0.5}^{N+0.5} P_{\text{cont.}}\left(X\right) \mathrm{d}X \tag{S8}$$

In principle, such a discretization would also allow for domains containing no rows ($X \in [0; 0.5[$), but with a low probability. To simplify the simulation algorithm, we also limited the maximum number of rows in a single domain to 8. This ensures that within the transfer width a considerable number of domains is present.

Following the STM data shown in the main paper, we set $\mu = 4$ and $\sigma = 1 - e^{-1} \approx 0.63$ with e being the Euler number. The result of this simulation is shown in Figure S6f. Compared to the regular structure some additional spots are quenched. At the same time the spots wash out along the $\left[1\bar{1}0\right]$ direction. Therefore, the agreement with the experimental data is certainly better as for the regular arrangement of the rows.

In the main paper, we argued that there should be left- and right-handed domains of standing upright molecules present on the surface. Such arrangements would allow either the naphtyl or the NCAuCl group to face the surface. It also affects the spacings between the rows and even lead to nonequivalent domain walls L-R versus R-L. Certainly, gold has the largest atom form factor f among the elements contained in the (NapNC)AuCl molecules.[8] The gold atoms are highly asymmetrically bound within the molecule – see Figure 1 of the main paper. Steric repulsion of the napthyl groups should lead to a large ‘phase shift’ between adjacent domains, which we cannot confirm with our simulations. Therefore, we suggest that the (NapNC)AuCl molecules are facing downward to the surface with the NCAuCl group and that the naphtyl group is exposed to the vacuum. The main contribution in the diffraction pattern would therefore originate from the napthyl-group. This is supported by the fact that the molecules consists of 10 carbon atoms but just one gold atom. In addition, the inelastic mean free path of the electron at the used energy is pretty much limited to one atomic layer according to the universal curve published by Seah and Dench,[9] so that the electron hardly

reaches the gold atom underneath the napthyl groups.

Such limitation of the penetration length of the impinging electrons would also attenuate all contributions of the substrate surface to the diffraction pattern. In agreement with this, the photoelectron spectra shown in Figure S1 for coverages Θ above one monolayer, do not show any contribution from the substrate. We can even infer that the electrons used in the LEED experiment have a shorter penetration length because their energy is higher. Consequently, the observed intensities originate solely from the diffraction at the adsorbate layer.

References

(1) Derry, G. N.; Kern, M. E.; Worth, E. H. Recommended values of clean metal surface work functions. *J. Vac. Sci. Technol. A: Vac. Surf. Films* **2015**, *33*, 060801.

(2) van Hove, M. A.; Weinberg, W. H.; Chan, C.-M. *Low-Energy Electron Diffraction Experiment, Theory and Surface Structure Determination*; Springer Berlin Heidelberg, 1986; Chapter Kinematic LEED Theory and Its Limits, pp 91–144.

(3) Moritz, W.; Wolf, D. Multilayer distortion in the reconstructed (110) surface of Au. *Surf. Sci. Lett.* **1985**, *163*, L655–L665.

(4) Moritz, W.; Wolf, D. Structure determination of the reconstructed Au(110) surface. *Surf. Sci.* **1979**, *88*, L29–L34.

(5) Hobbollahi, E.; List, M.; Redhammer, G.; Zabel, M.; Monkowius, U. Structural and photophysical characterisation of gold(I) complexes bearing naphthyl chromophores. *Inorg. Chem. Commun.* **2016**, *65*, 24–27.

(6) Pukite, P. R.; Lent, C. S.; Cohen, P. I. Diffraction from stepped surfaces. *Surf. Sci.* **1985**, *161*, 39–68.

(7) Wagner, T.; Fritz, D. R.; Zimmerleiter, R.; Zeppenfeld, P. On the microscopic structure of a nominal Ag(441) surface. *Surf. Sci.* **2017**, *661*, 77–82.

(8) Ibers, J. A. Atomic scattering amplitudes for electrons. *Acta Crystallogr.* **1958**, *11*, 178–183.

(9) Seah, M. P.; Dench, W. A. Quantitative electron spectroscopy of surfaces: A standard data base for electron inelastic mean free paths in solids. *Surf. Interface Anal.* **1979**, *1*, 2–11.
